# Regulatory layers of robustness of CLAVATA-WUSCHEL feedback system in stem cell homeostasis

**DOI:** 10.3389/fpls.2026.1777664

**Published:** 2026-03-11

**Authors:** Vincent E. Cerbantez-Bueno, Paddy J. Sheils, G. Venugopala Reddy

**Affiliations:** Department of Botany and Plant Sciences, University of California, Riverside, Riverside, CA, United States

**Keywords:** Arabidopsis, CLAVATA1, CLAVATA3, receptor kinase, WUSCHEL

## Abstract

Spatio-temporal regulation of gene expression and cellular behavior is critical for ensuring developmental robustness in all multicellular organisms. The mechanisms regulating robustness are often multilayered, with underlying processes occurring at different spatio-temporal scales. Pluripotent stem cells are maintained in SAMs despite periodic differentiation of stem-cell progeny into all above-ground organs, which together form much of the biomass on Earth. Shoot apical meristems (SAMs) are often exposed to variable growth conditions in nature; therefore, stem cell maintenance must be robust to withstand these changes. We focus our review on the mechanisms that regulate the robustness of the central CLAVATA (CLV)-WUSCHEL (WUS) feedback system, highlighting insights from new studies that integrate biological experiments with mathematical modeling. These studies have revealed the importance of WUS concentration-dependent transcription of *CLV3* involving cis-regulatory module and WUS-binding cofactors, CLV3 peptide processing and modifications, multiple signals converging on *WUS* transcriptional regulation, and WUS protein in regulating its subcellular partitioning, diffusion between adjacent cells, and degradation. We discuss mechanisms that could provide robustness to each of these processes and how their integration could provide tissue-level robustness in stem cell maintenance.

## Introduction

**Figure 1:**
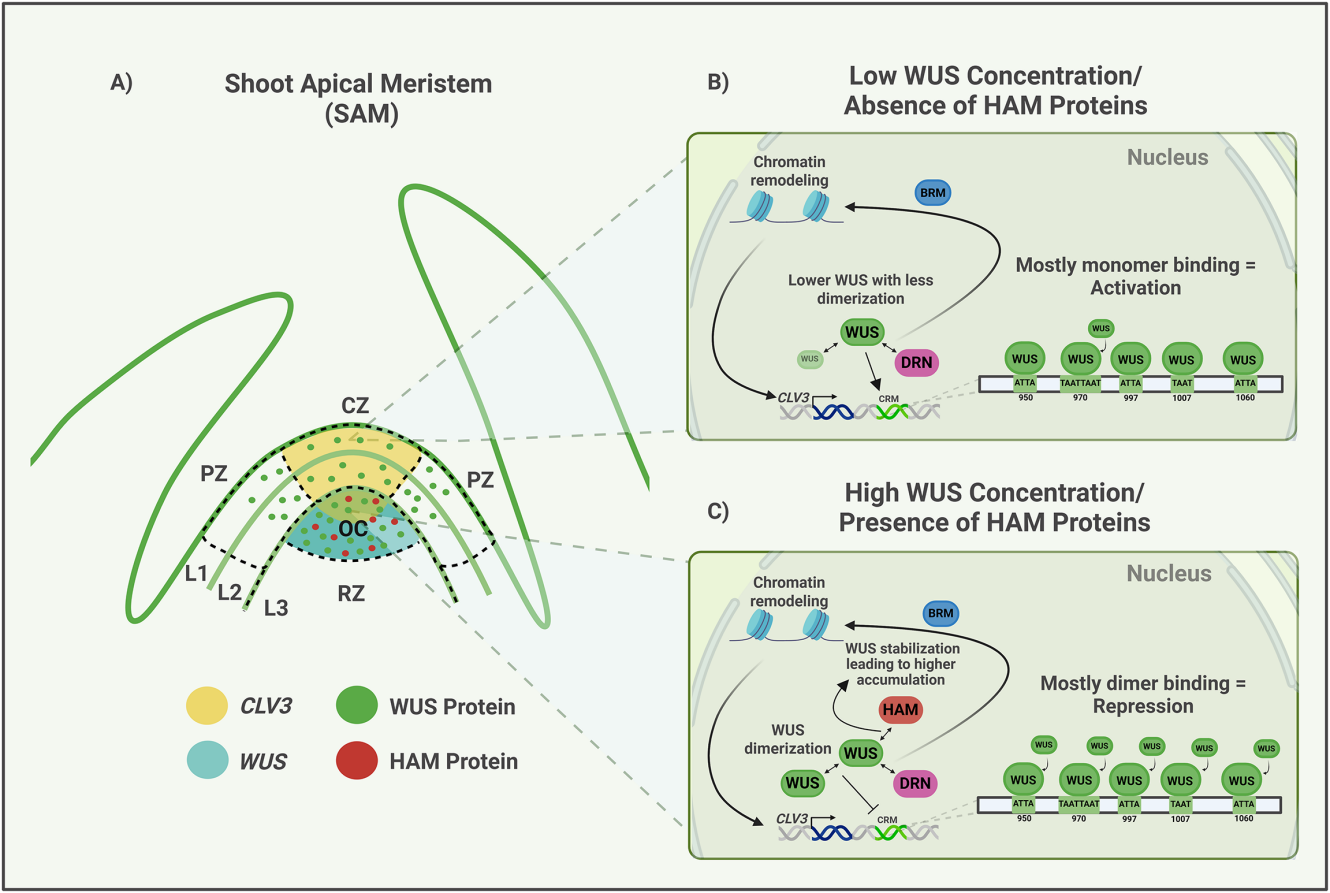
Regulation of *CLV3* transcription. **(A)** Shoot Apical Meristem (SAM) representation, showing its functional domains and cell layers, as well as the expression domains and/or protein localization of *CLV3*, WUS and HAM. CZ, Central Zone; PZ, Peripheral Zone; OC, Organizing Center; RZ, Rib Zone. L1, Layer 1; L2, Layer 2; L3, Layer 3. **(B)** Model showing SAM outer layers dynamics for *CLV3* regulation. At low WUS concentration and in absence of HAM proteins, the diffused WUS forms less dimers and binds the cis-elements mostly as a monomer and activates *CLV3* transcription. **(C)** Model showing SAM inner layers dynamics for *CLV3* regulation. In presence of HAM proteins, HAM interacts with WUS, stabilizing and promoting higher WUS accumulation, which leads to dimer formation and mostly repression of *CLV3*. In both scenarios, WUS interacts with DRN and recruits BRM to remodel chromatin and regulate *CLV3* expression. Created in BioRender. https://BioRender.com/gvcb5gu.

Under favorable conditions, plants can maintain indeterminate growth due to the continuous proliferation of stem cells in the plant meristems. The Shoot Apical Meristem (SAM) in flowering plants harbors the stem cells that differentiate into all aerial tissues (flowers, fruits, stem). The SAM is organized in functional domains and three clonally distinct cell layers (Steeves and Sussex, 1989). The central zone (CZ) harbors stem cells that divide relatively slowly, and their progeny are displaced laterally into the peripheral zone (PZ), where they divide about 3 times faster and differentiate into organ primordia (Reddy et al., 2004). The outer layers (L1 and L2) are maintained as monolayers by repeated anticlinal cell divisions, forming new cell walls perpendicular to the SAM surface, which will give rise to the epidermal (L1) and subepidermal (L2) tissues. Cells in deeper regions form the third layer (L3), and they divide in both periclinal (parallel to the SAM surface) and anticlinal orientations, forming multiple cell layers (Steeves and Sussex, 1989; Gaillochet and Lohmann, 2015) The organizing center (OC) is located at the apical region of the L3 layers, while cells beneath it form the rib zone (RZ), which differentiates to generate the pith and stem tissues (Shi and Vernoux, 2019) (**Figure 1A**). The balance between cell proliferation and bidirectional differentiation maintains the size and shape of the SAM. SAM development and maintenance must be robust, which could be defined as its capacity to maintain homeostasis among functional domains and cell layers despite periodic differentiation and changing environmental conditions (Aichinger et al., 2012). Several genetic pathways involved in chemical and mechanical signaling have been implicated in stem cell maintenance and SAM development (Hamant et al., 2008; Barton, 2010; Ma et al., 2019; Demesa-Arevalo et al., 2025; Lindsay et al., 2024). Here, we review multiple layers of regulation of the CLV-WUS feedback system and discuss their importance in providing robustness to stem cell homeostasis.

The isolation and characterization of mutants of larger and smaller SAMs have provided insights into cell-cell communication mechanisms among resident cells. In *Arabidopsis*, enlarged meristem phenotypes were found to result from mutations in *CLAVATA* (*CLV*) genes (Clark et al., 1995, 1997; Kayes and Clark, 1998; Fletcher et al., 1999; Hirakawa, 2021). Among them, *CLV3* encodes a 96-amino acid (aa) secreted peptide (Fletcher et al., 1999), which is further processed into a biologically active 12- or 13-aa peptide (Kondo et al., 2006). The biological activity of the CLV3 peptide is enhanced by posttranslational modifications such as hydroxylation and arabinosylation (Kondo et al., 2006; Ohyama et al., 2009). CLV3, in fact, belongs to a large peptide family referred to as CLAVATA3-LIKE/ENDOSPERM SURROUNDING REGION (CLE) family, which acts as signaling molecules in various plant pathways (Cock and McCormick, 2001; Fletcher, 2020). CLV3 peptide can be perceived by the founding member CLV1 and other related plasma membrane receptors (Clark et al., 1997; Kayes and Clark, 1998; Müller et al., 2008; Ogawa et al., 2008; Kinoshita et al., 2010; Nimchuk et al., 2011). CLV1 belongs to the plasma membrane receptor kinase family with characteristic extracellular leucine-rich repeats (LRRs) (Clark et al., 1997; Ogawa et al., 2008). CLV1 transmits the CLV3 signal by activating its intracellular kinase domain, leading to downstream phosphorylation cascades (Stone et al., 1998; Betsuyaku et al., 2010). Mutations in either of these genes lead to stem cell overproliferation, indicating that the CLV pathway restricts stem cell specification and SAM size.

CLV3 activating the CLV1 and related receptor kinase pathways restricts *WUSCHEL* (*WUS*) expression, which encodes a homedomain transcription factor (TF), expressed in the OC (Mayer et al., 1998; Schoof et al., 2000). The SAMs fail to develop in *wus* mutants, while the *WUS* expression expands in *clv* mutants (Mayer et al., 1998; Fletcher, 2018). Conversely, overexpression of *WUS* causes enlargement of the CZ and SAMs, showing that *WUS* is both necessary and sufficient for stem cell specification and SAM growth, which is restricted by CLV signaling (Mayer et al., 1998). Moreover, WUS activates *CLV3* expression, suggesting that these two genes mutually regulate each other to maintain stem cell homeostasis (Schoof et al., 2000; Brand et al., 2000). The *WUS* expressed in the OC must act non-cell autonomously to specific stem cells and activate *CLV3* in the CZ. This puzzle was solved much later, when a study showed that the WUS protein synthesized in the OC moves into adjacent cells, including the CZ, where it binds the *CLV3* gene directly (Yadav et al., 2011). Here, WUS also directly represses several differentiation-promoting TFs, preventing premature differentiation of stem cell progeny (Yadav et al., 2013). Recent studies have focused on the detailed regulation of CLV3 and WUS, both at transcriptional and post-transcriptional levels in *Arabidopsis* and other systems. We review these studies in the context of the regulation of robustness in stem cell homeostasis and SAM growth dynamics. Specifically, we synthesize findings on topics covering *CLV3* transcriptional regulation; CLV3 peptide maturation and post-translational processing; CLV3-mediated receptor signaling through CLV1 and related receptor families; regulation of *WUS* transcription and post-translational control of the WUS protein. We highlight emerging themes from quantitative approaches to explain how robustness of a dynamic system is achieved through redundancy, compensation, spatial feedback, and multi-step regulatory safeguards.

## Regulation of *CLV3* transcription

Most of the mechanistic insights into *CLV3* regulation come from *Arabidopsis thaliana* studies; however, comparative studies indicate that the logic of *CLV3* regulation is broadly conserved across angiosperms, with lineage-specific modifications (Suzaki et al., 2008; Xu et al., 2015). In *Arabidopsis*, *CLV3* is expressed mainly in the apical stem cell layers (L1 and L2) of the CZ and in few apical cells of the OC (Fletcher et al., 1999) (**Figure 1A**). However, in inner stem cell layers (L3/OC) *CLV3* expression is repressed. This transcriptional regulation is one of the main regulations that sustains WUS-CLV3 feedback loop and consequently, the robustness of SAM maintenance. Although other transcriptional regulators are implicated in *CLV3* regulation (Brand et al., 2002; Rodriguez et al., 2024; Luo et al., 2024), its expression in the CZ largely depends on WUS. *WUS* is expressed in the OC, and WUS protein moves into the CZ, likely through plasmodesmata (PD) (Yadav et al., 2011; Daum et al., 2014), forming a concentration gradient (**Figure 1A**). Though the WUS protein accumulates broadly in the SAM, *CLV3* is predominantly activated in the CZ, while its expression becomes strongly repressed in the OC (Yadav et al., 2011). Two competing models have been proposed to explain the spatial expression of *CLV3* (**Figures 1B, C**). The first one involves WUS concentration-dependent switching between activation and repression of *CLV3*, which is based on the following observations. WUS has been shown to bind a cis-regulatory module (CRM) comprising five cis-elements with varying binding affinities as monomers at lower concentrations and as dimers at higher concentrations (Perales et al., 2016). Incremental mutation of these cis-elements decreased *CLV3* expression in the CZ (L1 and L2 layers), and increased expression in the L3/OC. Taken together, these observations support a concentration-dependent model in which high WUS levels relatively favor dimers formation leading to stronger repression (**Figure 1C**), whereas lower WUS levels relatively favor monomeric binding to promote *CLV3* activation (**Figure 1B**). *CLV3* expression detected in very few apical cells of the OC suggests that monomeric WUS binding still occurs there (Yadav et al., 2011), albeit at reduced levels which could be due to the fluctuations in WUS protein levels which is regulated by multiple processes discussed later in this review. Consistent with this model, increasing the WUS-binding affinity of the cis-element led to dimerization at lower WUS concentrations and decreased *CLV3* expression in outer L1 and L2 cell layers of the CZ (Rodriguez et al., 2022). Moreover, increasing WUS concentration also repressed *CLV3* in all cell layers (Plong et al., 2021). Whereas partial depletion of WUS elevated *CLV3* and severe depletion decreased *CLV3* expression. This shows that *CLV3* expression is regulated within a window of WUS levels, with an activation threshold below which *CLV3* is not expressed and a repression threshold above which *CLV3* is repressed. To understand the significance of this regulation, a stochastic mathematical model was developed to capture the binding and unbinding dynamics of WUS to cis-elements and subsequent interactions with the transcriptional activation machinery (Rodriguez et al., 2022). The experimentally calibrated model revealed that the number of cis-elements, their WUS-binding affinities, and the spacing between cis-elements combine to influence cooperativity among WUS-bound cis-elements, thereby strongly repressing *CLV3* in inner cell layers and activating it in outer cell layers, thereby providing robustness to *CLV3* expression. Understanding how individual cis-elements adjust their binding affinities to fine-tune co-operativity levels to changing WUS concentrations requires coupling a stochastic WUS-binding *CLV3* transcription model with CLV3 signaling model regulating the WUS protein gradient.

The second competing model involves the spatial expression and localization of HAIRY MERISTEM (HAM) family proteins, HAM1 and HAM2, in the OC (Zhou et al., 2018) (**Figure 1A**). HAM proteins have been shown to physically interact with the C-terminus of WUS (Zhou et al., 2014). In *ham1 ham2* double mutants, *CLV3* expression has been observed to be downregulated in outer cell layers of the CZ but misexpressed in the OC (Han et al., 2020). However, a recent study has shown elevated *CLV3* expression across all cell layers, including the L1 and L2 layers of the CZ, and in cells of the OC of *ham1 ham2* double mutants. A similar *CLV3* expression pattern was observed in the genetic context involving the deletion of the HAM-binding domain of the WUS protein (Rodriguez et al., 2024). These results suggest that HAM proteins may act locally in the OC and non-cell autonomously to repress *CLV3* expression even in the outer cell layers of the CZ. WUS protein has been shown to diffuse broadly and accumulate at much lower levels in *ham* mutants (Rodriguez et al., 2024), revealing that HAM proteins stabilize WUS and inhibit its diffusion (**Figures 1B, C**). A similar higher *CLV3* activation was observed upon ectopic misexpression of *WUS*, which destabilized the WUS protein, leading to much lower levels (Perales et al., 2016). Therefore, the HAM-mediated regulation of maintenance of higher nuclear levels of the WUS protein provides a unified explanation for both the cell autonomous function in the OC and the non-cell autonomous function in the CZ in repressing *CLV3* expression. While HAM proteins provide robustness to the WUS protein gradient, whether they facilitate recruitment of other factors, such as TOPLESS (TPL), a component of the Histone Deacetylase (HDAC) complex, which also binds to the C-terminus of WUS (Causier et al., 2011), to tether WUS to chromatin, leading to nuclear retention, needs to be tested.

As in *Arabidopsis*, *CLV3-like CLE* genes are predominantly activated in the CZ of the SAM in other angiosperms (Suzaki et al., 2008; Li B. et al., 2025). However, in several species, including *Solanum lycopersicum* (Xu et al., 2015), *Glycine max* (Wong et al., 2013), and *Lotus japonicus* (Okamoto et al., 2011), *CLV3* expression has been shown to overlap with that of *WUS* expression in the OC. Whether these differences are due to the cis-element divergence needs further investigation. A recent study has shown that *CLV3* regulatory sequences are less conserved in *Solanum lycopersicum*, except for a small 27-bp stretch that aligns with the WUS-binding CRM of *Arabidopsis* (Ciren et al., 2024). Interestingly, the 27-bp stretch contains only one lower-affinity WUS-binding cis-element found in *Arabidopsis* CRM (Perales et al., 2016). Whether the loss of a higher-affinity cis-element and maintenance of only one cis-element in *Solanum*, as opposed to five cis-elements in *Arabidopsis*, contributed to *CLV3* expression in the OC needs to be tested. Therefore, comparative studies involving other species may reveal insights into the evolution of regulatory sequences leading to a better understanding of the cis-regulatory contribution to robustness of *CLV3* expression and SAM maintenance.

How the WUS concentration-dependent binding of WUS to the same cis-elements translates into activation and repression of *CLV3* is not understood. One possibility is that WUS dimers recruit the co-repressor TPL to mediate repression, whereas WUS monomers interact with co-activators, such as DORNROSCHEN (DRN), which binds to the homodimerization domain of WUS (**Figures 1B, C**) (Luo et al., 2024). The resulting DRN-WUS heterodimers recruit the chromatin remodeler BRAHAMA (BRM), leading to nucleosome depletion and increased chromatin accessibility at *the CLV3* regulatory region (Luo et al., 2024) (**Figures 1B, C**). Although chromatin regulation has not been fully incorporated into the WUS-CLV3 model, chromatin accessibility constitutes an essential regulatory layer that coordinates the precision of gene expression in tissues, including SAM. In addition, *CLV3* is responsive to hormones and other environmental cues, which indirectly influence its transcription. For example, auxin acts through ARF5/MONOPTEROS to repress a positive regulator of *CLV3*, the DRN (Luo et al., 2018), and light conditions affect auxin distribution, impacting *CLV3* expression (Pfeiffer et al., 2016). Recent work has shown that temperature modulates the spatial domain and activity of *CLV3* expression (Wenzl and Lohmann, 2023).

## Post-translational processing of CLV3 peptide

**Figure 2:**
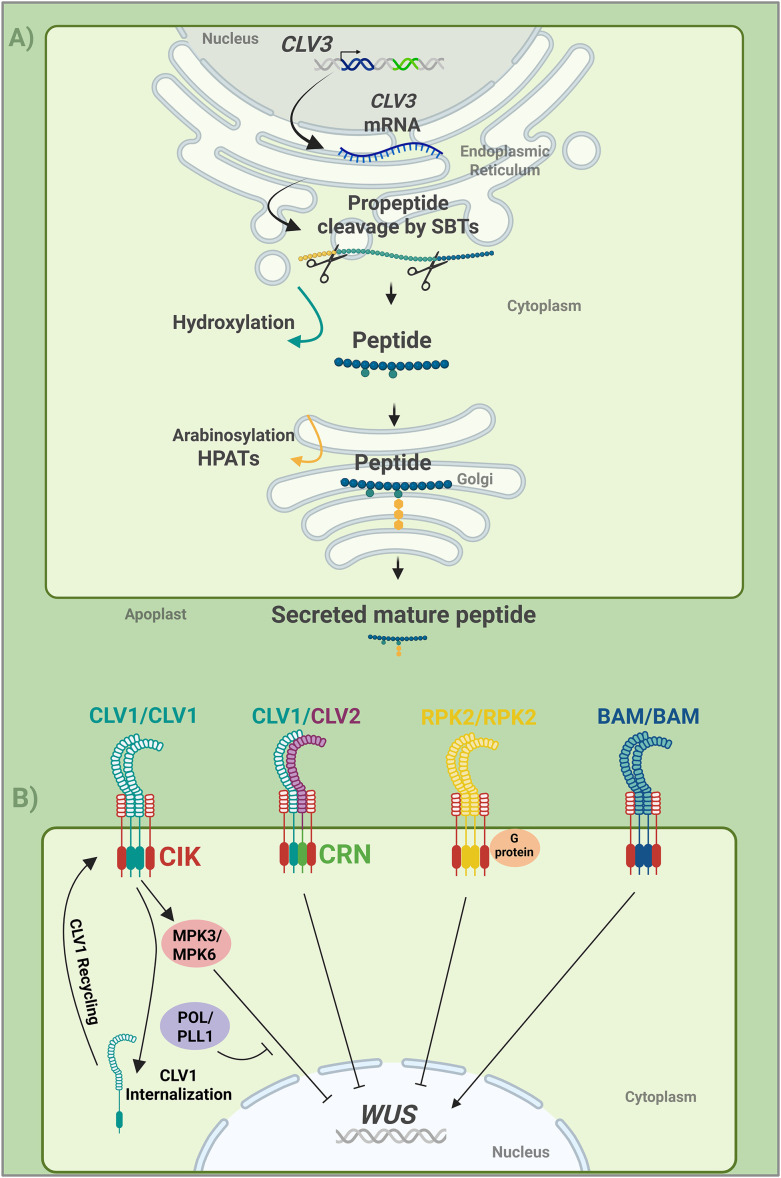
CLV3 peptide processing and signaling. **(A)** Model showing how CLV3 peptide is produced and perceived in the SAM. CLV3 is synthesized as a precursor propeptide, then processed and chemically modified before being secreted as a mature peptide. These modifications include cleavage by subtilin-like serine proteases (SBT) enzymes to generate the 12–13 amino acid peptide, which is then hydroxylated and arabinosylated at Golgi by Hyp O-arabinosyltransferases (HPATs) enzymes. The mature peptide is secreted into apoplast. **(B)** Model showing CLV1 and related receptors that mediate the peptide signaling to repress or activate *WUS* expression. After the peptide binding, the receptor triggers the signaling that subsequently represses or activate *WUS;* CLV1 signaling is negatively regulated by POL or PLL1. The model includes the CLV1 dynamics after CLV3 perception, such as internalization and receptor recycling. Created in BioRender. https://BioRender.com/qc24foz.

In *Arabidopsis*, *CLV3* mRNA encodes a 96–amino acid polypeptide precursor that undergoes multiple post-translational processing steps to generate the mature, bioactive CLV3 peptide (Stührwohldt and Schaller, 2019) (**Figure 2A**). Each of these maturation steps contributes to the robustness of CLV3-mediated signaling output and is highly conserved across other plant species, including tomato, legumes, and soybean (Wang et al., 2025). CLV3 belongs to the CLE protein family, which is characterized by having an N-terminal secretion signal, a highly conserved C-terminal CLE domain, and a variable middle domain (Fletcher, 2020). CLE propeptides precursors are proteolytically cleaved at the N- and C- termini by subtilin-like serine proteases (SBTs) to generate the mature 12–13 amino acid biologically active peptide (Betsuyaku et al., 2010; Stührwohldt et al., 2020; Nakagami et al., 2024) (**Figure 2A**). Specifically, two sites have been identified for CLV3 precursor cleavage: one before Met39 and another one before Arg70 (Xu et al., 2013). This cleaving process precedes the next steps of maturation. Hydroxylation and O-arabinosylation are essential and conserved post-translational modifications found in several CLE peptides, which enhance their full biological function (Ohyama et al., 2009; Shinohara and Matsubayashi, 2013; Wang et al., 2025). Many CLE peptides are known to be modified with L-arabinose residues on hydroxyproline (Hyp), including CLV3 (Xu et al., 2013). In *Arabidopsis*, two forms of CLV3 peptides have been found: a 12 amino acid peptide with Hyp residues in the 4^th^ and 7^th^ positions, and a 13 amino acid peptide that, with Hyp at the 4^th^ position and tri-arabinosylated Hyp at the 7^th^ position, contains an additional histidine residue at the 13^th^ position (Kondo et al., 2006; Ohyama et al., 2009; Nakagami et al., 2024). The tri-arabinosylation is catalyzed by the Hyp O-arabinosyltransferases (HPATs) located at the Golgi (Ogawa-Ohnishi et al., 2013) (**Figure 2A**). Notably, environmental conditions such as temperature can influence CLV3 arabinosylation, as demonstrated in tomato (Li Y. et al., 2025). Cold temperatures affect the transport of sugar into shoot apices, which is critical to maintain the glucose and arabinose supply required for CLV3 arabinosylation and proper meristem function. Whether the role of O-arabionosylation is strictly necessary for the CLV3 peptide function has been debated for years (Yamaguchi et al., 2016; Petersen et al., 2021). However, recent studies confirm that tri-arabinosylation is a shared feature that increases CLE peptide bioactivity (Nakagami et al., 2024). CLE peptide maturation follows a path from the endoplasmic reticulum (ER) to the Golgi, before being secreted to the apoplast (Stührwohldt et al., 2020) (**Figure 2A**). Although these mechanisms are well established, the precise sequence of events and the complete set of enzymes involved in CLV3 maturation remain incompletely characterized (Wang et al., 2025). Recently developed labels to track the CLV3 peptide (Zhou et al., 2024) may allow us to map the complete pathway and modifications that CLV3 undergoes before becoming a secreted, fully active, and stable peptide. The apoplast stability of the peptide in the extracellular space depends on the previous maturation steps, which act as a key regulatory layer that provides robustness to the signaling output. CLE peptides have been demonstrated to accumulate extracellularly and remain biologically active after secretion (Nakagami et al., 2024). This supports a model in which the control of maturation and secretion steps ensures a ligand bioactivity, stability, and effective signaling range in the apoplast.

Biological activity of CLE peptides in SAM control has been established in several species, including Arabidopsis (Song et al., 2022), rice (Suzaki et al., 2008), and maize (Fletcher, 2018). Studies have suggested that synthesis and processing leading to the biologically active peptide may be limited, as SAMs can tolerate very high levels of *CLV3* expression (Müller et al., 2006; Yadav et al., 2011). A recent study, in which *WUS* was overexpressed, destabilized WUS despite very high *CLV3* expression (Plong et al., 2021). However, the addition of a biologically active CLE peptide led to a stable WUS accumulation, suggesting that peptide production pathways could be limited, adding a fail-safe mechanism to handle high *CLV3* expression.

## CLV3 signaling

Once the CLE peptides are fully mature, they are secreted into the apoplast, a universal feature of CLE signaling, including CLV3 (Stührwohldt and Schaller, 2019). Their stability and signaling range can be influenced by environmental factors, such as temperature, light, and nutrients (Bashyal et al., 2024). The CLE peptides are perceived by the extracellular leucine-rich-repeat (LRR) domains of plasma membrane localized receptor-like kinases (RLK) complexes, which interact with co-receptors. Each RLK consists of the LRR extracellular domain, a transmembrane domain, and an intracellular serine/threonine kinase domain that triggers the signaling by phosphorylating downstream targets (Shiu and Bleecker, 2001; Hohmann et al., 2017). The receptor complex related to CLV3 signaling includes, but is not limited to, the LRR-RLK CLAVATA1 (CLV1), the receptor-like protein CLAVATA2 (CLV2), the membrane-associated pseudokinase CORYNE (CRN), which acts in concert with CLV2, and the CLAVATA3 INSENSITIVE RECEPTOR KINASES (CIKs), which work as co-receptors (Müller et al., 2008; Bleckmann et al., 2010; Hu et al., 2018;, Bashyal et al., 2024) (**Figure 2B**). Notably, the core elements of the CLV signaling system have been reported as a conserved feature in land plants (Whitewoods et al., 2018).

CLV1 has a complete LRR-RLK structure and remains central to CLV3 perception. CLV1 perceives CLV3 through its extracellular LRR domain and activates intracellular signaling via its cytoplasmic serine/threonine kinase domain, which undergoes ligand-dependent autophosphorylation (Ogawa et al., 2008; Song et al., 2022). Although most of the knowledge on CLV1 function comes from Arabidopsis studies, its role has been reported to be conserved across vascular and nonvascular plants (Hazak and Hardtke, 2016; Mirzaei et al., 2017; Whitewoods et al., 2018). CLV1 forms homodimers and interacts with the CIK family co-receptors to propagate downstream phosphorylation events (Hu et al., 2018) (**Figure 2B**). CLV3-CLV1 interaction not only activates the kinase activity but also induces ligand-dependent changes in CLV1 abundance and subcellular trafficking (Nimchuk et al., 2011). Upon CLV3 binding, a portion of CLV1 receptors is internalized via endocytosis from the plasma membrane, thereby modulating receptor availability (Nimchuk et al., 2011; Stührwohldt and Schaller, 2019) (**Figure 2B**). This serves as a negative feedback mechanism that buffers the intensity of CLV3-mediated signals. The internalized CLV1 can be recycled back to the plasma membrane or targeted for degradation (Nimchuk et al., 2011). This would allow the cell to fine-tune receptor levels in response to fluctuating concentrations of CLV3 peptide.

CLV2 forms a heterodimer with CRN and forms a complex that is associated with CLV1 (Bleckmann et al., 2010) (**Figure 2B**). Since CLV2 lacks the serine/threonine kinase domain, its function requires other factors. Despite being genetically essential for CLV3 signaling, this complex does not perceive CLV3 peptide (Song et al., 2022). Instead, it cooperates with additional LRR-RLKs, such as members of the CIKs co-receptor family, to form a functional signaling complex that mediates CLV3-dependent regulation of meristem homeostasis (Somssich et al., 2016; Hu et al., 2018) (**Figure 2B**). After CLV3 perception, CLV1 initiates phosphorylation cascades that activate targets that regulate *WUSCHEL* expression. CLV3 signaling rapidly activates the MAPK cascade (**Figure 2B**), particularly MPK3 and MPK6, through the MKK4/MKK5 modules (Lee et al., 2019) (**Figure 2B**). However, there are protein phosphatases that act as negative regulators to balance the signals. For example, POLTERIGEIST (POL) and its homolog PLL1 dephosphorylate key components of the CLV1 signaling pathway (Song et al., 2006; Somssich et al., 2016; Lee et al., 2019) (**Figure 2B**). The activity of these phosphatases is essential for maintaining *WUS* levels, hence the stem cell population.

Although CLV3-CLV1 remains the primary mechanism for stem cell maintenance in SAM, other mechanisms have been shown to provide robustness to this system. The CLV signaling pathway may be branched, and RECEPTOR-LIKE KINASE 2 (RPK2) contributes to CLV3 signaling and comprises a signaling module with G protein β subunit1 AGB1 (Kinoshita et al., 2010; Ishida et al., 2014). Though RPK2 does not directly interact with the CLV3 peptide, it regulates its signaling through the interaction with CIK proteins (Somssich et al., 2016; Hu et al., 2018). RPK2 can interact with itself or additional receptors, such as BARELY ANY MERISTEM (BAM), which have been implicated in the SAM maintenance (DeYoung et al., 2006; Somssich et al., 2016) (**Figure 2B**). *BAM* genes are expressed in the PZ, where they restrict *CLV1* expression, while CLV1 reciprocally represses *BAM* in the CZ. Despite this spatial separation, they act partially redundant, enhancing the robustness of signaling (DeYoung et al., 2006; Deyoung and Clark, 2008; Nimchuk et al., 2015). Recent studies have established that BAM receptors mediate distinct, context-dependent CLE signaling pathways across tissues (Cornelis and Hazak, 2025). The CLE40-mediated signaling in activating *WUS* transcription is discussed in the next section. Though CLV3 is the most important CLE peptide in maintaining stem cell activity, recent reports have shown that other CLE family members can compensate for CLV3 activity (Dao et al., 2022). CLE16 and CLE17 contribute to a compensatory signaling mechanism that partially substitutes for CLV3 activity when CLV3 levels are reduced or absent. This compensation suggests that multiple CLE peptides collaborate in the CLV–WUS feedback loop, through ensuring robustness in stem-cell regulation even when the CLV3 signal is compromised.

## Regulation of *WUS* transcription

**Figure 3:**
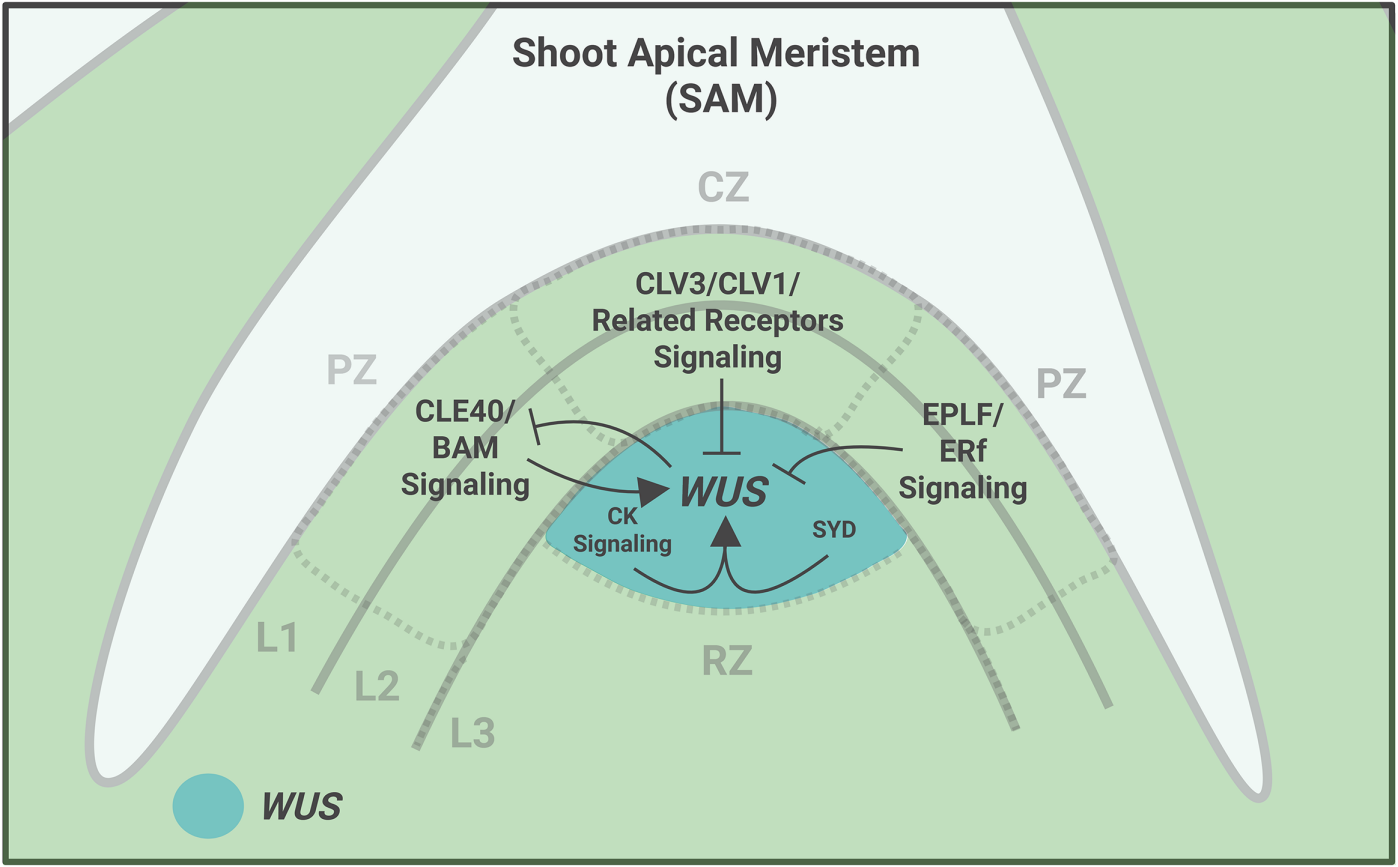
Regulation of *WUS* expression. Model showing the signaling dynamics in the Shoot Apical Meristem (SAM) that regulate *WUS* expression and its restriction to the organizing center (OC). *WUS* expression in the OC is positively regulated by cytokinin signaling (CK) and CLE40/BAM signaling originating from the peripheral zone (PZ). Simultaneously, *WUS* is negatively regulated by CLV3/CLV1 and associated receptors signaling module originating from the central zone (CZ). EPLF/ERf signaling originating from the PZ restricts *WUS* expression in the OC. Created in BioRender. https://BioRender.com/i87699o.

CLV3 signaling restricted *WUS* expression to the OC (**Figure 3**), and the loss of CLV3 function led to an upward expansion of the *WUS* expression domain toward the L2 layer in *clv3* mutants (Schoof et al., 2000). In vegetative SAMs of *clv3* mutants, *WUS* expression has been shown to expand into the RZ along with an increase in levels (Uzair et al., 2024). Additionally, *clv3* mutants show increased *WUS* mRNA accumulation in OC cells compared to wild type. These findings indicate that CLV3 negatively regulates *WUS* expression levels and position based on the developmental context. As *CLV3* is predominantly expressed in CZ layers (L1 and L2), the CLV3 peptide could be highly produced here. However, *CLV3* expression overlaps with *WUS* expression in a few apically located cells in the OC, which may produce the functional CLV3 peptide, raising the possibility of CLV3 acting as an autocrine signal in regulating *WUS* expression levels (Uzair et al., 2024; Shpak and Uzair, 2025). Analysis using the mathematical model suggested that CLV3 regulates *WUS* expression levels primarily through autocrine signaling. Whether CLV3 also functions as an autocrine signal in inhibiting the nuclear export of the WUS protein to regulate its concentration gradient (discussed in the next section) needs further investigation. However, new lines of investigations on the spatial localization of functional CLV3 peptide in SAMs and the cell autonomous function of CLV3 peptide are required to support the autocrine signaling model. In addition to regulating the *WUS* expression levels in the OC, it is necessary to spatially restrict its expansion along the radial axis. This role is redundantly fulfilled by the ERECTA family receptors (ERf), which are activated by the EPIDERMAL PATTERNING FACTOR LIKE (EPFL) ligands expressed in the SAM periphery (Kosentka et al., 2019; Zhang et al., 2021; Uzair et al., 2024; Liu et al., 2020) (**Figure 3**). Though receptor kinase signaling has been implicated in repression of *WUS* transcription, the identity of transcriptional repressors and the mechanisms of transcriptional repression have remained elusive.

CLV3-WUS and EPLF-ERf signaling represent the negative feedback loop that restricts spatial activation and levels of *WUS* transcription; however, positive regulation is required to maintain *WUS* expression. The cytokinins produced in the CZ diffuse into the OC and activate *WUS* transcription (Gordon et al., 2009; Shpak and Uzair, 2025) (**Figure 3**). Cytokinins are perceived first by the AHK receptors, which are expressed in the inner layers of the SAM (Adibi et al., 2016). Upon binding to their receptors, they activate a multistep phosphorelay involving Histidine Phosphotransferase (AHP) proteins, which translocate to the nucleus and activate the Type B Response Regulators (ARR). The ARRs bind directly to the *WUS* promoter to activate its expression (Meng et al., 2017). More recent research indicates that cytokinin-response transcriptional regulators differ in their spatial and temporal contributions to *WUS* regulation, enabling fine-tuning of *WUS* expression across the shoot apical meristem (Demesa-Arevalo et al., 2025). Notably, WUS could positively affect its own expression by enhancing sensitivity to cytokinin through repression of A-type ARRs (Leibfried et al., 2005). However, the CK responsiveness was not compromised in *wus* null mutants, suggesting WUS-independent regulation (Snipes et al., 2018). Recent study has shown that CLE40 peptide originating from the PZ cells acts through BAM1 receptors to promote *WUS* expression in the OC (Schlegel et al., 2021) (**Figure 3**). WUS, in turn, negatively regulates *CLE40* expression, establishing a feedback loop between PZ and OC (Schlegel et al., 2021) (**Figure 3**). CLE40-BAM1 pathway acting antagonistically to the central CLV3-CLV1 pathway in regulating *WUS* expression shows another layer of inter-regional communication among functional domains in regulating stem cell homeostasis.

Additionally, several specific and general chromatin regulators and histone chaperones are involved in confining *WUS* expression in the OC (Shen and Xu, 2009; Singh et al., 2020). For example, the SNF2 chromatin-remodeling ATPase SPLAYED (SYD) is required for maintenance of the stable stem cell pool by positive regulation of *WUS* expression in the OC of the SAM (Kwon et al., 2005) (**Figure 3**). Environmental factors also modulate *WUS* level and spatial distribution. For example, light promotes *WUS* induction and heat stress represses its transcription (Pfeiffer et al., 2016; Liu et al., 2024).

## Regulation of the WUS protein gradient

A precise regulation of the WUS protein gradient is critical in regulating stem cell homeostasis and SAM growth. WUS has been shown to prevent premature differentiation of the immediate stem cell progeny by directly repressing the expression of many differentiation-promoting genes (Yadav et al., 2013). The spatial distribution of the WUS protein is influenced by intrinsic protein features and layer-specific extrinsic signals. Many of the transcriptional regulatory domains, such as the N-terminal homeodomain, which also acts as one of the homodimerization domains (HOD1), the central homodimerization domain (HOD2), together restrict WUS protein diffusion (Rodriguez et al., 2016). While the C-terminally located WUS-Box, HAM-binding domain, and the ERF-linked amphiphilic repression (EAR)-like domain also regulate nucleus-cytoplasmic (N-C) partitioning, diffusion, and stability. Here, we discuss how extrinsic signals, such as CLV3 and the plant hormone cytokinin (CK), act on intrinsic signals to provide robustness to stem cell homeostasis.

**Figure 4:**
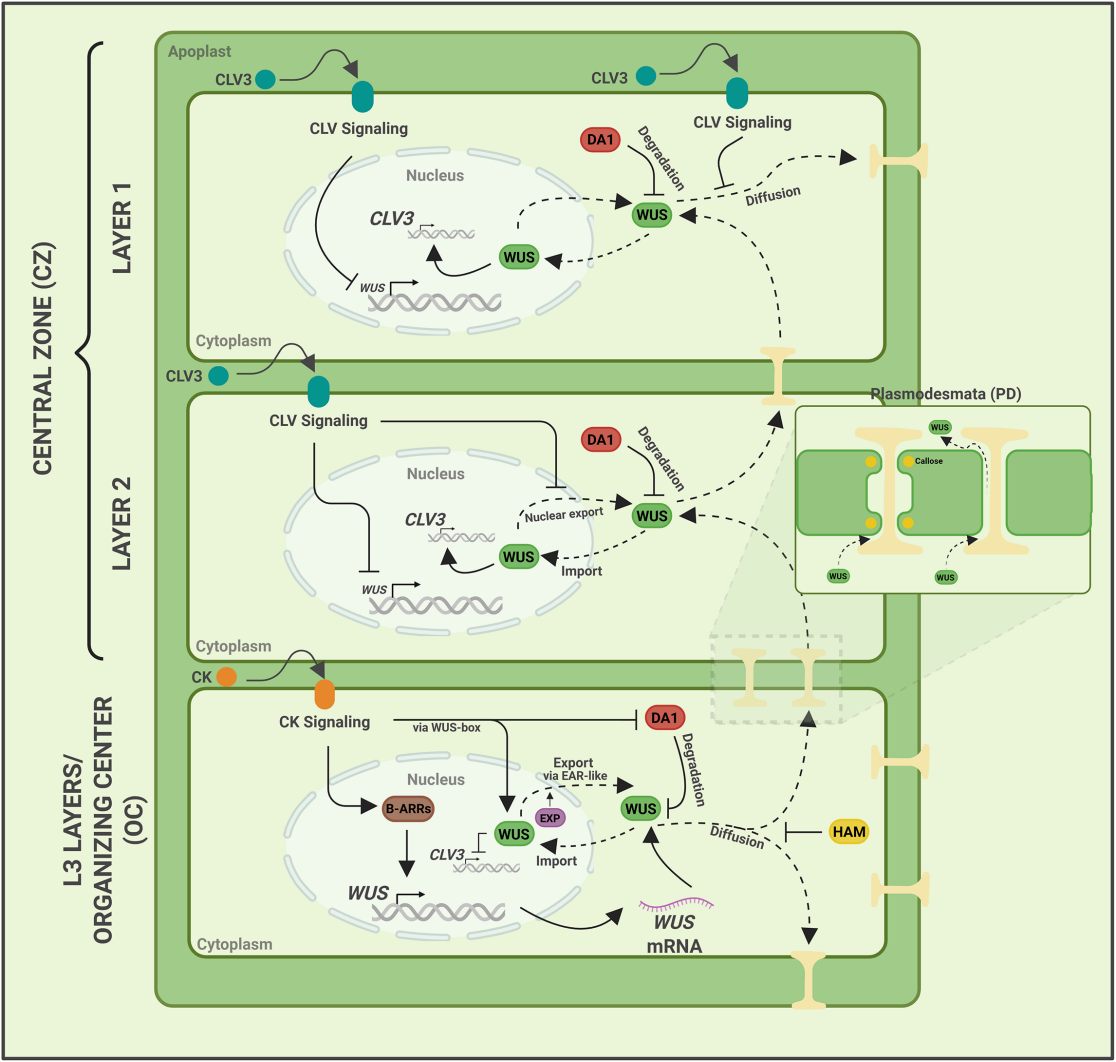
Regulation of the WUS protein gradient. Model showing how WUS is activated or repressed in the different layers of the SAM, and the mechanisms regulating WUS protein gradient. *WUS* expression is activated by cytokinin (CK) signaling in inner layers and repressed by CLV3 signaling in outer layers. WUS protein gradient is regulated by mechanisms that regulate the N-C partitioning in which CLV3 offsets nuclear export and CK acts in nuclear retention, the diffusion depends on the N-C ratio. WUS export into the cytoplasm is mediated by the EAR-like domain which is also required for its degradation in the cytoplasm. DA1 mediates WUS degradation, which in turn is downregulated by the CK signaling. Dotted lines represent WUS movement. Created in BioRender. https://BioRender.com/p4y0i31.

After its translation in the OC, the WUS homeodomain transcription factor moves into adjacent cells likely via cytoplasmic bridges called plasmodesmata (Demesa-Arevalo et al., 2025) (**Figure 4**). Imaging studies using 2xeGFP-WUS reporters revealed that WUS diffusion is limited, resulting in a stable protein gradient rather than uniform distribution (Rodriguez et al., 2016). One mechanism contributing to this constraint is regulation of plasmodesmata permeability: callose deposition at plasmodesmata limits WUS movement and altered callose synthase activity changes the spatial extent of WUS diffusion within the meristem (Daum et al., 2014) (**Figure 4**). A study aimed at explaining the development of multilocular fruit under cold temperatures has shown that a higher callose accumulation induced by cold restricts WUS intercellular mobility into the CZ, leading to reduced *CLV3* and increased *WUS* synthesis (Wu et al., 2023).

The intercellular movement and regulation of the WUS protein gradient are shaped by N-C partitioning, which, in turn, is regulated by the competing processes of nuclear retention and nuclear export (Rodriguez et al., 2016) (**Figure 4**). Mutating the WUS-box led to a dramatic accumulation of WUS in the cytoplasm. On the contrary, the EAR-like domain mutations revealed a higher nuclear accumulation. At the same time, the double mutants of WUS-Box and EAR-like domain revealed a higher nuclear accumulation similar to the EAR-like domain mutants. These observations suggested that the WUS-box promotes nuclear retention while the EAR-like domain mediates nuclear export. A later study showed that EXPORTIN proteins interact with the EAR-like domain to mediate nuclear export of WUS (Plong et al., 2021).

To achieve the spatial gradient of WUS, nuclear retention and nuclear export must be regulated to varying degrees across different cell layers. CK signaling active in the L3 layers of the RZ has been shown to act on the WUS-box, the nuclear retention signal, in promoting nuclear accumulation and stability (Snipes et al., 2018). CLV3 signaling has been shown to offset nuclear export, especially in the outer cell layers, to regulate the N-C ratio and thereby prevent excessive diffusion into neighboring cells and overproliferation of the SAMs observed in *clv3* mutants. A recent study showing differentiation of the CZ in *clv3* mutants could be due to unregulated, excessive nuclear export, leading to lower nuclear levels of WUS (Rambaud-Lavigne et al., 2024), suggesting that CLV3-mediated signaling is required to balance N-C partitioning to maintain requisite WUS levels in the nucleus and to control spatial diffusion. To uncouple CLV3-mediated *WUS* transcription from its post-translational role in offsetting nuclear export, and to understand their contribution to robustness, a continuous partial differentiation equation (PDE) signaling model was developed (Plong et al., 2021). The *in silico* manipulation of the experimentally calibrated model revealed that dual regulation is critical for maintaining the WUS protein gradient by forming a feedback loop with WUS-concentration-dependent transcriptional activation and repression of *CLV3* (Plong et al., 2021). As WUS protein levels rise, *CLV3* is repressed, leading to excessive nuclear export; the resulting lower WUS levels activate CLV3, which inhibits nuclear export, thus maintaining stability via feedback regulation. In essence, this feedback regulation, involving dual control, maintains nuclear WUS levels despite variations in *WUS* expression.

The WUS protein gradient is also regulated by degradation (**Figure 4**). A higher nuclear accumulation has been shown to stabilize WUS, suggesting a link between WUS concentration and stability, and that the protein exported from the nucleus is degraded in the cytoplasm (Perales et al., 2016; Plong et al., 2021). It has also been found that the same EAR-like domain that functions as a nuclear export signal is required for destabilizing WUS in the cytoplasm (Plong et al., 2021). A recent study shows that DA1, a ubiquitin-regulated peptidase, interacts directly with WUS protein to cleave it at specific sites and destabilize it (Cui et al., 2024). This prevents a higher WUS accumulation outside the OC, thereby sharpening the gradient. Significantly, the plant hormone CK active in the RZ has been shown to repress DA1 activity, thereby promoting WUS stability in this region, thus contributing to the gradient (Cui et al., 2024). This mechanism creates a spatially restricted balance of WUS protein abundance. Taken together, layer-specific signals that act on subcellular processes regulate the WUS protein gradient. Moreover, the utilization of transcriptional regulatory domains, such as the WUS-box for nuclear retention and the EAR-like domain for nuclear export and degradation, establishes a link between regulation of protein concentration and transcription, forming a spatio-temporally coupled system that provides robustness to feedback regulation.

Although the intrinsic regulation of various subcellular processes has been well understood, the potential post-translational protein modifications underlying these processes remain limited. Proteomic analysis has identified phosphorylation sites on the WUS protein, suggesting regulation by kinase- based signaling pathways (Dory et al., 2016). These findings raise the possibility that MAP kinase signaling, including MPK3 downstream of CLV3 perception, directly modulates WUS through phosphorylation. Understanding the role of such modification in regulating the signal-dependent WUS stability, sub-cellular localization, or interactions with other proteins could provide additional insights into the regulation of the WUS gradient.

Although most mechanistic insight into the regulation of the WUS protein gradient comes from *Arabidopsis thaliana*, regulated regulatory frameworks appear to operate in other plant species. In rice, maize, tomato, and soybean, WUS and related homeobox genes show spatially restricted expression and function within CLE peptide-mediated signaling pathways that limit stem cell proliferation (Wong et al., 2013; Tanaka et al., 2015; Je et al., 2016; Somssich et al., 2016). However, in these species, evidence largely comes from transcript-level expression and genetic analysis, and direct visualization of WUS protein accumulation or gradients has not been well established. Non-cell autonomous signaling, receptor-mediated feedback, and protein stability are therefore inferred to contribute to meristem size control, although the relative importance of these mechanisms likely varies across species (Fletcher, 2018). Together, these observations suggest that the core principles of WUS-mediated stem cell regulation are conserved, while the underlying molecular implementations have diversified with meristem architecture.

## Future perspectives

The dynamic regulation of stem cell maintenance, growth, and differentiation in SAMs is controlled by several processes that govern the synthesis, processing, and signaling of the central players, WUS and CLV3, occurring at various time scales across subcellular, cellular, and tissue scales. Multiple layers of regulation have been found in *CLV3* transcription, CLV3 peptide maturation, *WUS* transcription, and WUS protein regulation. To integrate spatio-temporal events across multiple scales, it is essential to develop multiscale models. Quantitative analysis was performed using stochastic modeling of *CLV3* transcription, which enabled quantification of cooperativity among cis-elements and determinants of cooperativity (Rodriguez et al., 2022). PDE-based signaling models have been developed to study the mutual regulation of CLV3 and WUS (Plong et al., 2021), WUS-mediated repression of differentiation (Yadav et al., 2013), and ERECTA family (ERf) receptors (Uzair et al., 2024). Future work should couple these sub-models to generate hybrid models in which all aspects of the regulation can be manipulated, individually or in combination, and their effects analyzed quantitatively. This would involve developing a stochastic model of WUS-mediated repression of differentiation supported by *in vivo* data on cis-element manipulations and biochemical data on WUS binding to the cis-elements in repressed target genes. Since WUS accumulates at the lowest level in the lateral edges of the PZ, the repressed target genes may contain higher-affinity cis-elements similar to the ones described in structural analysis (Sloan et al., 2020), and/or a greater number of cis-elements within the CRM, where intervening spacing is optimally organized to facilitate better interaction among WUS bound to the cis-elements.

Another frontier is to go beyond implementing these hybrid models on a static SAM template and instead develop a growth model based on live imaging time-lapse data (Reddy et al., 2004; Willis et al., 2016). Agent-based growth models have been developed to explain how tissue geometry and the chemical signaling network regulate SAM gene expression (Gruel et al., 2016). However, the agent model represents cells as spheres without considering their actual shapes and geometry, which is critical for obtaining shared cell contact areas between adjacent cells to model PD distribution and diffusion fluxes between neighboring cells. Such models would enable analysis of causal relationships among cell division planes, PD distribution, and WUS diffusion, regulated by signals that are difficult to understand in experiments alone. As examples, it has been shown that CK promotes periclinal cell division, which could be relevant for generating multiple cell layers of the L3. Still, it could also affect WUS diffusion into the inner cell layers (Banwarth-Kuhn et al., 2022). *wus* mutants divide periclinally in the L2 layer, suggesting WUS is required for fostering anticlinal cell divisions in the L2 layer, which could impact WUS mobility into the L2 and the L1 layers. Similarly, downregulation of *CLV3* or misexpression of WUS promotes PZ cell division (Reddy and Meyerowitz, 2005; Yadav et al., 2010), suggesting that the WUS gradient could organize cell division rates, which in turn could impact WUS diffusion radially. In obtaining cell shapes and geometry, progress in image analysis is also critical for analyzing large amounts of time-lapse datasets by incorporating Artificial Intelligence/Machine Learning (AI/ML) approaches (Islam et al., 2025).

## References

[B1] Adibi M. Yoshida S. Weijers D. Fleck C. (2016). Centering the organizing center in the Arabidopsis thaliana shoot apical meristem by a combination of cytokinin signaling and self-organization. PloS One 11, e0147830. doi: 10.1371/JOURNAL.PONE.0147830, PMID: 26872130 PMC4752473

[B2] Aichinger E. Kornet N. Friedrich T. Laux T. (2012). Plant stem cell niches. Annu. Rev. Plant Biol. 63, 615–636. doi: 10.1146/annurev-arplant-042811-105555, PMID: 22404469

[B3] Banwarth-Kuhn M. Rodriguez K. Michael C. Ta C. K. Plong A. Bourgain-Chang E. . (2022). Combined computational modeling and experimental analysis integrating chemical and mechanical signals suggests possible mechanism of shoot meristem maintenance. PloS Comput. Biol. 18, e1010199. doi: 10.1371/JOURNAL.PCBI.1010199, PMID: 35727850 PMC9249181

[B4] Barton M. K. (2010). Twenty years on: The inner workings of the shoot apical meristem, a developmental dynamo. Dev. Biol. 341, 95–113. doi: 10.1016/j.ydbio.2009.11.029, PMID: 19961843

[B5] Bashyal S. Gautam C. K. Müller L. M. (2024). CLAVATA signaling in plant–environment interactions. Plant Physiol. 194, 1336–1357. doi: 10.1093/PLPHYS/KIAD591, PMID: 37930810 PMC10904329

[B6] Betsuyaku S. Takahashi F. Kinoshita A. Miwa H. Shinozaki K. Fukuda H. . (2010). Mitogen-activated protein kinase regulated by the CLAVATA receptors contributes to shoot apical meristem homeostasis. Plant Cell Physiol. 52, 14. doi: 10.1093/PCP/PCQ157, PMID: 20965998 PMC3023851

[B7] Bleckmann A. Weidtkamp-Peters S. Seidel C. A. M. Simon R. (2010). Stem cell signaling in Arabidopsis requires CRN to localize CLV2 to the plasma membrane. Plant Physiol. 152, 166–176. doi: 10.1104/PP.109.149930, PMID: 19933383 PMC2799354

[B8] Brand U. Fletcher J. C. Hobe M. Meyerowitz E. M. Simon R. (2000). Dependence of stem cell fate in Arabidopsis on a feedback loop regulated by CLV3 activity. Sci. (New York N.Y.) 289, 617–619. doi: 10.1126/SCIENCE.289.5479.617, PMID: 10915624

[B9] Brand U. Grünewald M. Hobe M. Simon R. (2002). Regulation of CLV3 expression by two homeobox genes in Arabidopsis. Plant Physiol. 129, 565. doi: 10.1104/PP.001867, PMID: 12068101 PMC161677

[B10] Causier B. Ashworth M. Guo W. Davies B. (2011). The TOPLESS interactome: A framework for gene repression in Arabidopsis. Plant Physiol. 158, 423. doi: 10.1104/PP.111.186999, PMID: 22065421 PMC3252085

[B11] Ciren D. Zebell S. Lippman Z. B. (2024). Extreme restructuring of cis-regulatory regions controlling a deeply conserved plant stem cell regulator. PloS Genet. 20 (3), e1011174. doi: 10.1371/JOURNAL.PGEN.1011174, PMID: 38437180 PMC10911594

[B12] Clark S. E. Running M. P. Meyerowitz E. M. (1995). CLAVATA3 is a specific regulator of shoot and floral meristem development affecting the same processes as CLAVATA1. Development 121, 2057–2067. doi: 10.1242/DEV.121.7.2057, PMID: 39484095

[B13] Clark S. E. Williams R. W. Meyerowitz E. M. (1997). The CLAVATA1 gene encodes a putative receptor kinase that controls shoot and floral meristem size in Arabidopsis. Cell 89, 575–585. doi: 10.1016/S0092-8674(00)80239-1, PMID: 9160749

[B14] Cock J. M. McCormick S. (2001). A large family of genes that share homology with CLAVATA3. Plant Physiol. 126, 939–942. doi: 10.1104/PP.126.3.939, PMID: 11457943 PMC1540125

[B15] Cornelis S. Hazak O. (2025). CLE pathways in plant development: recent advances and future perspectives. J. Exp. Bot. 76, 5748–5754. doi: 10.1093/JXB/ERAF321, PMID: 40660820 PMC12605765

[B16] Cui G. Li Y. Zheng L. Smith C. Bevan M. W. Li Y. (2024). The peptidase DA1 cleaves and destabilizes WUSCHEL to control shoot apical meristem size. Nat. Commun. 15, 4627. doi: 10.1038/s41467-024-48361-7, PMID: 38821962 PMC11143343

[B17] Dao T. Q. Weksler N. Liu H. M. H. Leiboff S. Fletcher J. C. (2022). Interactive CLV3, CLE16 and CLE17 signaling mediates stem cell homeostasis in the Arabidopsis shoot apical meristem. Dev. (Cambridge England) 149 (19). doi: 10.1242/DEV.200787, PMID: 36111520

[B18] Daum G. Medzihradszky A. Suzaki T. Lohmann J. U. (2014). A mechanistic framework for noncell autonomous stem cell induction in Arabidopsis. Proc. Natl. Acad. Sci. United States America 111, 14619–14624. doi: 10.1073/PNAS.1406446111, PMID: 25246576 PMC4210042

[B19] Demesa-Arevalo E. Narasimhan M. Simon R. (2025). Intercellular communication in shoot meristems. On: Wed. 64, 4. doi: 10.1146/annurev-arplant-070523, PMID: 38424066

[B20] DeYoung B. J. Bickle K. L. Schrage K. J. Muskett P. Patel K. Clark S. E. (2006). The CLAVATA1-related BAM1, BAM2 and BAM3 receptor kinase-like proteins are required for meristem function in Arabidopsis. Plant J. 45, 1–16. doi: 10.1111/j.1365-313X.2005.02592.x, PMID: 16367950

[B21] Deyoung B. J. Clark S. E. (2008). BAM receptors regulate stem cell specification and organ development through complex interactions with CLAVATA signaling. Genetics 180, 895–904. doi: 10.1534/genetics.108.091108, PMID: 18780746 PMC2567389

[B22] Dory M. Doleschall Z. Nagy S. K. Ambrus H. Mészáros T. Barnabás B. . (2016). Kinase-Associated Phosphoisoform Assay: a novel candidate-based method to detect specific kinase-substrate phosphorylation interactions in *vivo*. BMC Plant Biol. 16, 204. doi: 10.1186/S12870-016-0894-1, PMID: 27655033 PMC5031308

[B25] Fletcher J. C. (2018). The CLV-WUS stem cell signaling pathway: A roadmap to crop yield optimization. Plants 7 (4), 87. doi: 10.3390/PLANTS7040087, PMID: 30347700 PMC6313860

[B24] Fletcher J. C. (2020). Recent advances in Arabidopsis CLE peptide signaling. Trends Plant Sci. 25, 1005–1016. doi: 10.1016/j.tplants.2020.04.014, PMID: 32402660

[B23] Fletcher J. C. Brand U. Running M. P. Simon R. Meyerowitz E. M. Fletcher J. C. . (1999). Signaling of cell fate decisions by CLAVATA3 in Arabidopsis shoot meristems. Sci 283, 1911. doi: 10.1126/SCIENCE.283.5409.1911, PMID: 10082464

[B26] Gaillochet C. Lohmann J. U. (2015). The never-ending story: from pluripotency to plant developmental plasticity. Development 142, 2237–2249. doi: 10.1242/dev.117614, PMID: 26130755 PMC4510588

[B27] Gordon S. P. Chickarmane V. S. Ohno C. Meyerowitz E. M. (2009). Multiple feedback loops through cytokinin signaling control stem cell number within the Arabidopsis shoot meristem. Proc. Natl. Acad. Sci. United States America 106, 16529–16534. doi: 10.1073/PNAS.0908122106, PMID: 19717465 PMC2752578

[B28] Gruel J. Landrein B. Tarr P. Schuster C. Refahi Y. Sampathkumar A. . (2016). An epidermis-driven mechanism positions and scales stem cell niches in plants. Sci. Adv. 2, e1500989. doi: 10.1126/sciadv.1500989, PMID: 27152324 PMC4846443

[B30] Hamant O. Heisler M. G. Jönsson H. Krupinski P. Uyttewaal M. Bokov P. . (2008). Developmental patterning by mechanical signals in Arabidopsis. Science 322, 1650–1655. doi: 10.1126/science.1165594, PMID: 19074340

[B29] Han H. Geng Y. Guo L. Yan A. Meyerowitz E. M. Liu X. . (2020). The overlapping and distinct roles of HAM family genes in Arabidopsis shoot meristems. Front. Plant Sci. 11. doi: 10.3389/FPLS.2020.541968/BIBTEX PMC7498855 33013964

[B31] Hazak O. Hardtke C. S. (2016). CLAVATA 1-type receptors in plant development. J. Exp. Bot. 67, 4827–4833. doi: 10.1093/JXB/ERW247, PMID: 27340234

[B32] Hirakawa Y. (2021). CLAVATA3, a plant peptide controlling stem cell fate in the meristem. Peptides 142 (2021), 170579. doi: 10.1016/j.peptides.2021.170579, PMID: 34033873

[B33] Hohmann U. Lau K. Hothorn M. (2017). The structural basis of ligand perception and signal activation by receptor kinases. Annu. Rev. Plant Biol. 68, 109–137. doi: 10.1146/ANNUREV-ARPLANT-042916-040957, PMID: 28125280

[B34] Hu C. Zhu Y. Cui Y. Cheng K. Liang W. Wei Z. . (2018). A group of receptor kinases are essential for CLAVATA signalling to maintain stem cell homeostasis. Nat. Plants 4, 205–211. doi: 10.1038/S41477-018-0123-Z, PMID: 29581511

[B35] Ishida T. Tabata R. Yamada M. Aida M. Mitsumasu K. Fujiwara M. . (2014). Heterotrimeric G proteins control stem cell proliferation through CLAVATA signaling in Arabidopsis. EMBO Rep. 15, 1202–1209. doi: 10.15252/EMBR.201438660/FIGURES/4 25260844 PMC4253494

[B36] Islam M. S. Dutta A. Ta C. K. Rodriguez K. Michael C. Alber M. . (2025). DEGAST3D: learning deformable 3D graph similarity to track plant cells in unregistered time lapse images. IEEE Trans. Comput. Biol. Bioinf. 22, 343–354. doi: 10.1109/TCBBIO.2024.3525404, PMID: 40811238

[B37] Je B. Gruel J. Lee Y. K. Bommert P. Arevalo E. D. Eveland A. L. . (2016). Signaling from maize organ primordia via FASCIATED EAR3 regulates stem cell proliferation and yield traits. Nat. Genet. 48, 785–791. doi: 10.1038/ng.3567, PMID: 27182966

[B38] Kayes J. M. Clark S. E. (1998). CLAVATA2, a regulator of meristem and organ development in Arabidopsis. Dev. (Cambridge England) 125, 3843–3851. doi: 10.1242/DEV.125.19.3843, PMID: 9729492

[B39] Kinoshita A. Betsuyaku S. Osakabe Y. Mizuno S. Nagawa S. Stahl Y. . (2010). RPK2 is an essential receptor-like kinase that transmits the CLV3 signal in Arabidopsis. Dev. (Cambridge England) 137, 3911–3920. doi: 10.1242/DEV.048199, PMID: 20978082

[B40] Kondo T. Sawa S. Kinoshita A. Mizuno S. Kakimoto T. Fukuda H. . (2006). A plant peptide encoded by CLV3 identified by in *situ* MALDI-TOF MS analysis. Sci. (New York N.Y.) 313, 845–848. doi: 10.1126/SCIENCE.1128439, PMID: 16902141

[B41] Kosentka P. Z. Overholt A. Maradiaga R. Mitoubsi O. Shpak E. D. (2019). EPFL signals in the boundary region of the SAM restrict its size and promote leaf initiation. Plant Physiol. 179, 265–279. doi: 10.1104/PP.18.00714, PMID: 30409857 PMC6324244

[B42] Kwon C. S. Chen C. Wagner D. (2005). WUSCHEL is a primary target for transcriptional regulation by SPLAYED in dynamic control of stem cell fate in Arabidopsis. Genes Dev. 19, 992–1003. doi: 10.1101/gad.1276305, PMID: 15833920 PMC1080137

[B43] Lee H. Jun Y. S. Cha O. K. Sheen J. (2019). Mitogen-activated protein kinases MPK3 and MPK6 are required for stem cell maintenance in the Arabidopsis shoot apical meristem. Plant Cell Rep. 38, 311–319. doi: 10.1007/S00299-018-2367-5, PMID: 30552452 PMC6573032

[B44] Leibfried A. To J. P. C. Busch W. Stehling S. Kehle A. Demar M. . (2005). WUSCHEL controls meristem function by direct regulation of cytokinin-inducible response regulators. Nature 438, 1172–1175. doi: 10.1038/NATURE04270, PMID: 16372013

[B45] Li B. Liu W. Xu J. Huang X. Yang L. Xu F. (2025). Decoding maize meristems maintenance and differentiation: integrating single-cell and spatial omics. J. Genet. Genomics 52, 319–333. doi: 10.1016/J.JGG.2025.01.012, PMID: 39921079

[B46] Li Y. Wang J. Liang X. Wu S. Zhang J. Wu C. . (2025). STP2-mediated sugar transport in tomato shoot apices is critical for CLV3 arabinosylation and fruit locule development under low temperatures. Mol. Plant 18, 1014–1028. doi: 10.1016/j.molp.2025.05.002, PMID: 40336199

[B47] Lindsay P. Swentowsky K. W. Jackson D. (2024). Cultivating potential: Harnessing plant stem cells for agricultural crop improvement. Mol. Plant 17, 50–74. doi: 10.1016/j.molp.2023.12.014, PMID: 38130059

[B48] Liu Z. Shpak E. D. Hong T. (2020). A mathematical model for understanding synergistic regulations and paradoxical feedbacks in the shoot apical meristem. Comput. Struct. Biotechnol. J. 18, 3877–3889. doi: 10.1016/J.CSBJ.2020.11.017, PMID: 33335685 PMC7720093

[B49] Liu S. Wu H. Zhao Z. (2024). Heat stress-induced decapping of WUSCHEL mRNA enhances stem cell thermotolerance in Arabidopsis. Mol. Plant 17, 1820–1832. doi: 10.1016/j.molp.2024.10.011, PMID: 39468792

[B51] Luo L. Liu L. She L. Zhang H. Zhang N. Wang Y. . (2024). DRN facilitates WUS transcriptional regulatory activity by chromatin remodeling to regulate shoot stem cell homeostasis in Arabidopsis. PloS Biol. 22, e3002878. doi: 10.1371/JOURNAL.PBIO.3002878, PMID: 39514478 PMC11548754

[B50] Luo L. Zeng J. Wu H. Tian Z. Zhao Z. (2018). A molecular framework for auxin-controlled homeostasis of shoot stem cells in Arabidopsis. Mol. Plant 11, 899–913. doi: 10.1016/j.molp.2018.04.006, PMID: 29730265

[B52] Ma Y. Miotk A. Šutiković Z. Ermakova O. Wenzl C. Medzihradszky A. . (2019). WUSCHEL acts as an auxin response rheostat to maintain apical stem cells in Arabidopsis. Nat. Commun. 10, 5093. doi: 10.1038/s41467-019-13074-9, PMID: 31704928 PMC6841675

[B53] Mayer K. F. X. Schoof H. Haecker A. Lenhard M. Jürgens G. Laux T. (1998). Role of WUSCHEL in regulating stem cell fate in the Arabidopsis shoot meristem. Cell 95, 805–815. doi: 10.1016/S0092-8674(00)81703-1, PMID: 9865698

[B54] Meng W. J. Cheng Z. J. Sang Y. L. Zhang M. M. Rong X. F. Wang Z. W. . (2017). Type-B ARABIDOPSIS RESPONSE REGULATORs specify the shoot stem cell niche by dual regulation of WUSCHEL. Plant Cell 29, 1357–1372. doi: 10.1105/TPC.16.00640, PMID: 28576846 PMC5502443

[B55] Mirzaei S. Batley J. El-Mellouki T. Liu S. Meksem K. Ferguson B. J. . (2017). Neodiversification of homeologous CLAVATA1-like receptor kinase genes in soybean leads to distinct developmental outcomes. Sci. Rep. 7, 8878. doi: 10.1038/S41598-017-08252-Y, PMID: 28827708 PMC5566472

[B57] Müller R. Bleckmann A. Simon R. (2008). The receptor kinase CORYNE of Arabidopsis transmits the stem cell-limiting signal CLAVATA3 independently of CLAVATA1. Plant Cell 20, 934–946. doi: 10.1105/TPC.107.057547, PMID: 18381924 PMC2390746

[B56] Müller R. Borghi L. Kwiatkowska D. Laufs P. Simon R. (2006). Dynamic and compensatory responses of Arabidopsis shoot and floral meristems to CLV3 signaling. Plant Cell 18, 1188–1198. doi: 10.1105/tpc.105.040444, PMID: 16603652 PMC1456859

[B58] Nakagami S. Kajiwara T. Tsuda K. Sawa S. (2024). CLE peptide signaling in plant-microbe interactions. Front. Plant Sci. 15. doi: 10.3389/FPLS.2024.1481650/FULL PMC11538016 39507357

[B59] Nimchuk Z. L. Tarr P. T. Ohno C. Qu X. Meyerowitz E. M. (2011). Plant stem cell signaling involves ligand-dependent trafficking of the CLAVATA1 receptor kinase. Curr. Biol. 21, 345–352. doi: 10.1016/J.CUB.2011.01.039, PMID: 21333538 PMC3072602

[B60] Nimchuk Z. L. Zhou Y. Tarr P. T. Peterson B. A. Meyerowitz E. M. (2015). Plant stem cell maintenance by transcriptional cross-regulation of related receptor kinases. Dev. (Cambridge) 142, 1043–1049. doi: 10.1242/dev.119677, PMID: 25758219 PMC4360179

[B61] Ogawa M. Shinohara H. Sakagami Y. Matsubayash Y. (2008). Arabidopsis CLV3 peptide directly binds CLV1 ectodomain. Sci. (New York N.Y.) 319, 294. doi: 10.1126/SCIENCE.1150083, PMID: 18202283

[B62] Ogawa-Ohnishi M. Matsushita W. Matsubayashi Y. (2013). Identification of three hydroxyproline O-arabinosyltransferases in Arabidopsis thaliana. Nat. Chem. Biol. 9, 726–730. doi: 10.1038/nchembio.1351, PMID: 24036508

[B63] Ohyama K. Shinohara H. Ogawa-Ohnishi M. Matsubayashi Y. (2009). A glycopeptide regulating stem cell fate in Arabidopsis thaliana. Nat. Chem. Biol. 5, 578–580. doi: 10.1038/nchembio.182, PMID: 19525968

[B64] Okamoto S. Nakagawa T. Kawaguchi M. (2011). Expression and functional analysis of a CLV3-like gene in the model legume lotus aponicus. Plant Cell Physiol. 52, 1211–1221. doi: 10.1093/PCP/PCR071, PMID: 21652543

[B65] Perales M. Rodriguez K. Snipes S. Yadav R. K. Diaz-Mendoza M. Reddy G. V. (2016). Threshold-dependent transcriptional discrimination underlies stem cell homeostasis. Proc. Natl. Acad. Sci. United States America 113, E6298–E6306. doi: 10.1073/PNAS.1607669113;WGROUP:STRING:PUBLICATION PMC5068294 27671653

[B66] Petersen B. L. MacAlister C. A. Ulvskov P. (2021). Plant protein O-arabinosylation. Front. Plant Sci. 12. doi: 10.3389/FPLS.2021.645219, PMID: 33815452 PMC8012813

[B67] Pfeiffer A. Janocha D. Dong Y. Medzihradszky A. Schöne S. Daum G. . (2016). Integration of light and metabolic signals for stem cell activation at the shoot apical meristem. ELife 5, e17023. doi: 10.7554/ELIFE.17023, PMID: 27400267 PMC4969040

[B68] Plong A. Rodriguez K. Alber M. Chen W. Reddy G. V. (2021). CLAVATA3 mediated simultaneous control of transcriptional and post-translational processes provides robustness to the WUSCHEL gradient. Nat. Commun. 12, 6361. doi: 10.1038/s41467-021-26586-0, PMID: 34737298 PMC8569176

[B69] Rambaud-Lavigne L. Chatterjee A. Bovio S. Battu V. Lavigne Q. Gundiah N. . (2024). Heterogeneous identity, stiffness and growth characterise the shoot apex of Arabidopsis stem cell mutants. Dev. (Cambridge) 151 (11). doi: 10.1242/dev.202810, PMID: 38752444

[B70] Reddy G. V. Heisler M. G. Ehrhardt D. W. Meyerowitz E. M. (2004). Real-time lineage analysis reveals oriented cell divisions associated with morphogenesis at the shoot apex of Arabidopsis thaliana. Dev. (Cambridge England) 131, 4225–4237. doi: 10.1242/DEV.01261, PMID: 15280208

[B71] Reddy G. V. Meyerowitz E. M. (2005). Stem-cell homeostasis and growth dynamics can be uncoupled in the Arabidopsis shoot apex. Sci. (New York N.Y.) 310, 663–667. doi: 10.1126/SCIENCE.1116261, PMID: 16210497

[B73] Rodriguez K. Do A. Senay-Ara B. Perales M. Alber M. Chen W. . (2022). Concentration-dependent transcriptional switching through a collective action of cis-elements. Sci. Adv. 8 (32), eabo6157. doi: 10.1126/sciadv.abo6157, PMID: 35947668 PMC9365274

[B74] Rodriguez K. Kao L. Cerbantez-Bueno V. E. Delgadillo C. Nguyen D. Ullah S. . (2024). HAIRY MERISTEM proteins regulate the WUSCHEL protein levels in mediating CLAVATA3 expression. Physiol. Plant. 176 (5), e14505. doi: 10.1111/PPL.14505, PMID: 39221514

[B72] Rodriguez K. Perales M. Snipes S. Yadav R. K. Diaz-Mendoza M. Reddy G. V. (2016). DNA-dependent homodimerization, sub-cellular partitioning, and protein destabilization control WUSCHEL evels and spatial patterning. Proc. Natl. Acad. Sci. United States America 113, E6307–E6315. doi: 10.1073/PNAS.1607673113, PMID: 27671631 PMC5068338

[B75] Schlegel J. Denay G. Wink R. Pinto K. G. Stahl Y. Schmid J. . (2021). Control of Arabidopsis shoot stem cell homeostasis by two antagonistic CLE peptide signalling pathways. ELife 10. doi: 10.7554/ELIFE.70934, PMID: 34643181 PMC8594942

[B76] Schoof H. Lenhard M. Haecker A. Mayer K. F. X. Jürgens G. Laux T. (2000). The stem cell population of Arabidopsis shoot meristems is maintained by a regulatory loop between the CLAVATA and WUSCHEL genes. Cell 100, 635–644. doi: 10.1016/S0092-8674(00)80700-X, PMID: 10761929

[B77] Shen W. H. Xu L. (2009). Chromatin remodeling in stem cell maintenance in Arabidopsis thaliana. Mol. Plant 2, 600–609. doi: 10.1093/MP/SSP022, PMID: 19825642

[B78] Shi B. Vernoux T. (2019). Patterning at the shoot apical meristem and phyllotaxis. Curr. Topics Dev. Biol. 131, 81–107. doi: 10.1016/bs.ctdb.2018.10.003, PMID: 30612633

[B79] Shinohara H. Matsubayashi Y. (2013). Chemical synthesis of Arabidopsis CLV3 glycopeptide reveals the impact of hydroxyproline arabinosylation on peptide conformation and activity. Plant Cell Physiol. 54, 369–374. doi: 10.1093/PCP/PCS174, PMID: 23256149 PMC3589827

[B80] Shiu S.-H. Bleecker A. B. (2001). Plant receptor-like kinase gene family: diversity, function, and signaling. Science’s STKE: Signal Transduct. Knowledge Environ. 2001, re22–re22. doi: 10.1126/STKE.2001.113.RE22, PMID: 11752632

[B81] Shpak E. D. Uzair M. (2025). WUSCHEL: The essential regulator of the Arabidopsis shoot Apical Meristem. Curr. Opin. Plant Biol. 85, 102739. doi: 10.1016/j.pbi.2025.102739, PMID: 40381531

[B82] Singh S. Singh A. Singh A. Yadav S. Bajaj I. Kumar S. . (2020). Role of chromatin modification and remodeling n stem cell regulation and meristem maintenance in Arabidopsis. J. Exp. Bot. 71, 778–792. doi: 10.1093/jxb/erz459, PMID: 31793642

[B83] Sloan J. Hakenjos J. P. Gebert M. Ermakova O. Gumiero A. Stier G. . (2020). Structural basis for the complex DNA binding behavior of the plant stem cell regulator WUSCHEL. Nat. Commun. 11, 2223. doi: 10.1038/s41467-020-16024-y, PMID: 32376862 PMC7203112

[B84] Snipes S. A. Rodriguez K. DeVries A. E. Miyawaki K. N. Perales M. Xie M. . (2018). Cytokinin stabilizes WUSCHEL by acting on the protein domains required for nuclear enrichment and transcription. PloS Genet. 14, e1007351. doi: 10.1371/JOURNAL.PGEN.1007351, PMID: 29659567 PMC5919686

[B85] Somssich M. Je B. Simon R. Jackson D. (2016). CLAVATA-WUSCHEL signaling in the shoot meristem. Dev. (Cambridge England) 143, 3238–3248. doi: 10.1242/DEV.133645, PMID: 27624829

[B86] Song X. F. Hou X. L. Liu C. M. (2022). CLE peptides: critical regulators for stem cell maintenance in plants. Planta 255, 5. doi: 10.1007/S00425-021-03791-1, PMID: 34841457

[B87] Song S. K. Lee M. M. Clark S. E. (2006). POL and PLL1 phosphatases are CLAVATA1 signaling intermediates required for Arabidopsis shoot and floral stem cells. Development 133, 4691–4698. doi: 10.1242/DEV.02652, PMID: 17079273

[B88] Steeves T. A. Sussex I. M. (1989). Patterns in plant development (2nd ed.). (Cambridge: Cambridge University Press), 388.

[B89] Stone J. M. Trotochaud A. E. Walker J. C. Clark S. E. (1998). Control of meristem development by CLAVATA1 receptor kinase and kinase-associated protein phosphatase interactions. Plant Physiol. 117, 1217–1225. doi: 10.1104/PP.117.4.1217, PMID: 9701578 PMC34886

[B91] Stührwohldt N. Ehinger A. Thellmann K. Schaller A. (2020). Processing and formation of bioactive CLE40 peptide are controlled by posttranslational proline hydroxylation. Plant Physiol. 184, 1573. doi: 10.1104/PP.20.00528, PMID: 32907884 PMC7608152

[B90] Stührwohldt N. Schaller A. (2019). Regulation of plant peptide hormones and growth factors by post-translational modification. Plant Biol. (Stuttgart Germany) 21 Suppl 1, 49–63. doi: 10.1111/PLB.12881, PMID: 30047205

[B92] Suzaki T. Yoshida A. Hirano H. Y. (2008). Functional diversification of CLAVATA3-related CLE proteins in meristem maintenance in rice. Plant Cell 20, 2049. doi: 10.1105/TPC.107.057257, PMID: 18676878 PMC2553609

[B93] Tanaka W. Ohmori Y. Ushijima T. Matsusaka H. Matsushita T. Kumamaru T. . (2015). Axillary meristem formation in rice requires the WUSCHEL ortholog TILLERS ABSENT1. Plant Cell 27, 1173–1184. doi: 10.1105/tpc.15.00074, PMID: 25841039 PMC4558701

[B94] Uzair M. Urquidi Camacho R. A. Liu Z. Overholt A. M. DeGennaro D. Zhang L. . (2024). An updated model of shoot apical meristem regulation by ERECTA family and CLAVATA3 signaling pathways in Arabidopsis. Dev. (Cambridge) 151, dev202870. doi: 10.1242/DEV.202870/VIDEO-3 PMC11234387 38814747

[B95] Wang S. Liang Y. Jiang Y. Wang S. Liang Y. Jiang Y. (2025). Plant CLE peptides functions, challenges, and future prospects. Plant Hormones 1, 0–0. doi: 10.48130/PH-0025-0006. E006.

[B96] Wenzl C. Lohmann J. U. (2023). 3D imaging reveals apical stem cell responses to ambient temperature. Cells Dev. 175, 203850. doi: 10.1016/J.CDEV.2023.203850, PMID: 37182581

[B97] Whitewoods C. D. Cammarata J. Nemec Venza Z. Sang S. Crook A. D. Aoyama T. . (2018). CLAVATA was a genetic novelty for the morphological innovation of 3D growth in land plants. Curr. Biol. 28, 2365. doi: 10.1016/J.CUB.2018.05.068, PMID: 30033333 PMC6089843

[B98] Willis L. Refahi Y. Wightman R. Landrein B. Teles J. Huang K. C. . (2016). Cell size and growth regulation in the Arabidopsis thaliana apical stem cell niche. Proc. Natl. Acad. Sci. United States America 113, E8238–E8246. doi: 10.1073/PNAS.1616768113, PMID: 27930326 PMC5187701

[B99] Wong C. E. Singh M. B. Bhalla P. L. (2013). Spatial expression of CLAVATA3 in the shoot apical meristem suggests it is not a stem cell marker in soybean. J. Exp. Bot. 64, 5641. doi: 10.1093/JXB/ERT341, PMID: 24179098 PMC3871822

[B100] Wu J. Sun W. Sun C. Xu C. Li S. Li P. . (2023). Cold stress induces malformed tomato fruits by breaking the feedback loops of stem cell regulation in floral meristem. New Phytol. 237, 2268–2283. doi: 10.1111/NPH.18699, PMID: 36564973

[B102] Xu C. Liberatore K. L. Macalister C. A. Huang Z. Chu Y. H. Jiang K. . (2015). A cascade of arabinosyltransferases controls shoot meristem size in tomato. Nat. Genet. 47, 784–792. doi: 10.1038/NG.3309, PMID: 26005869

[B101] Xu T. T. Song X. F. Ren S. C. Liu C. M. (2013). The sequence flanking the N-terminus of the CLV3 peptide is critical for its cleavage and activity in stem cell regulation in Arabidopsis. BMC Plant Biol. 13, 225. doi: 10.1186/1471-2229-13-225, PMID: 24369789 PMC3878228

[B103] Yadav R. K. Perales M. Gruel J. Girke T. Jönsson H. Venugopala Reddy G. (2011). WUSCHEL protein movement mediates stem cell homeostasis in the Arabidopsis shoot apex. Genes Dev. 25, 2025–2030. doi: 10.1101/GAD.17258511, PMID: 21979915 PMC3197201

[B104] Yadav R. K. Perales M. Gruel J. Ohno C. Heisler M. Girke T. . (2013). Plant stem cell maintenance involves direct transcriptional repression of differentiation program. Mol. Syst. Biol. 9, MSB20138. doi: 10.1038/MSB.2013.8/TABLES/1 PMC3658276 23549482

[B105] Yadav R. K. Tavakkoli M. Reddy G. V. (2010). WUSCHEL mediates stem cell homeostasis by regulating stem cell number and patterns of cell division and differentiation of stem cell progenitors. Development 137, 3581–3589. doi: 10.1242/DEV.054973, PMID: 20876644

[B106] Yamaguchi Y. L. Ishida T. Sawa S. (2016). CLE peptides and their signaling pathways in plant development. J. Exp. Bot. 67 (16), 4813–4826. doi: 10.1093/jxb/erw208, PMID: 27229733

[B107] Zhang L. Gennaro D. Lin G. Chai J. Shpak E. D. (2021). ERECTA family signaling constrains CLAVATA3 and WUSCHEL to the center of the shoot apical meristem. Dev. (Cambridge England) 148 (5). doi: 10.1242/DEV.189753, PMID: 33593817

[B108] Zhou Y. Liu X. Engstrom E. M. Nimchuk Z. L. Pruneda-Paz J. L. Tarr P. T. . (2014). Control of plant stem cell function by conserved interacting transcriptional regulators. Nature 517, 377–380. doi: 10.1038/nature13853, PMID: 25363783 PMC4297503

[B109] Zhou Y. Yan A. Han H. Li T. Geng Y. Liu X. . (2018). Hairy meristem with wuschel confines clavata3 expression to the outer apical meristem layers. Science 361, 502–506. doi: 10.1126/science.aar8638, PMID: 30072538 PMC6095697

[B1000] Zhou Y. Zheng J. Yang H. Han H. (2024). A novel toolbox to record CLE peptide signaling. Front. Plant Sci. 15, 1468763. doi: 10.3389/fpls.2024.1468763, PMID: 39206038 PMC11349659

